# Successful Patient-Derived Organoid Culture of Gynecologic Cancers for Disease Modeling and Drug Sensitivity Testing

**DOI:** 10.3390/cancers13122901

**Published:** 2021-06-10

**Authors:** Jianling Bi, Andreea M. Newtson, Yuping Zhang, Eric J. Devor, Megan I. Samuelson, Kristina W. Thiel, Kimberly K. Leslie

**Affiliations:** 1 Department of Obstetrics and Gynecology, University of Iowa, Iowa City, IA 52242, USA; jianling-bi@uiowa.edu (J.B.); andreea-newtson@uiowa.edu (A.M.N.); yuping-zhang@uiowa.edu (Y.Z.); eric-devor@uiowa.edu (E.J.D.); kristina-thiel@uiowa.edu (K.W.T.); 2 Holden Comprehensive Cancer Center, University of Iowa, Iowa City, IA 52242, USA; 3 Department of Pathology, University of Iowa, Iowa City, IA 52242, USA; megan-samuelson@uiowa.edu

**Keywords:** patient-derived organoids (PDOs), endometrial cancer, ovarian cancer, drug sensitivity testing, personalized medicine

## Abstract

**Simple Summary:**

In the conventional treatment of gynecologic malignancies, most patients receive similar ‘one-size-fits-all’ treatment. However, it is increasingly clear that standard therapies do not work in every patient, and it would be very helpful to have pretreatment predictive assays to provide more personalized regimens. In this study, we describe the routine, successful establishment of patient-derived organoid models (PDOs) of endometrial and ovarian cancer tissues from consenting patients and provide an example of how information from drug screening in PDOs may be a useful predictor of patient response to therapy.

**Abstract:**

Developing reliable experimental models that can predict clinical response before treating the patient is a high priority in gynecologic cancer research, especially in advanced or recurrent endometrial and ovarian cancers. Patient-derived organoids (PDOs) represent such an opportunity. Herein, we describe our successful creation of 43 tumor organoid cultures and nine adjacent normal tissue organoid cultures derived from patients with endometrial or ovarian cancer. From an initial set of 45 tumor tissues and seven ascites fluid samples harvested at surgery, 83% grew as organoids. Drug sensitivity testing and organoid cell viability assays were performed in 19 PDOs, a process that was accomplished within seven days of obtaining the initial surgical tumor sample. Sufficient numbers of cells were obtained to facilitate testing of the most commonly used agents for ovarian and endometrial cancer. The models reflected a range of sensitivity to platinum-containing chemotherapy as well as other relevant agents. One PDO from a patient treated prior to surgery with neoadjuvant trastuzumab successfully predicted the patient’s postoperative chemotherapy and trastuzumab resistance. In addition, the PDO drug sensitivity assay identified alternative treatment options that are currently used in the second-line setting. Our findings suggest that PDOs could be used as a preclinical platform for personalized cancer therapy for gynecologic cancer patients.

## 1. Introduction

Ovarian cancer is the fifth leading cause of death of women from cancer worldwide [1]. Due to the lack of symptoms during the early development of ovarian cancer, patients are usually diagnosed at an advanced stage and have the lowest average five-year survival rate (46%) among all patients with gynecologic malignancies [2]. In 2020, over 21,000 women were diagnosed with ovarian cancer and approximately 14,000 deaths occurred from this disease in the United States [3]. Endometrial cancer is the most common gynecologic malignancy. Although many patients present at an early stage and enjoy a good prognosis, patients with advanced or recurrent endometrial cancer have a very low five-year survival rate (<20%). In 2020, over 65,000 women were diagnosed with endometrial cancer in the United States, and nearly 13,000 women died from this disease [3]. Additionally, endometrial cancer is one of only a few cancers for which the incidence and mortality have been increasing year after year since the 1970s [4].

Current standard treatments for ovarian and endometrial cancer are very similar and use a combination of surgery and chemotherapy [5,6]. Radiation and hormone therapies are two additional methods used to treat endometrial cancer, but for high-grade serous ovarian cancers and high-risk endometrial cancers, chemotherapy with the doublet of a platinum compound and a taxane is typically recommended [7,8]. Although most patients with advanced disease respond well to initial treatment, the majority develop recurrent disease and become resistant to chemotherapy. For recurrent and platinum-resistant disease, additional cytotoxic agents such as gemcitabine, topotecan, doxorubicin and liposomal doxorubicin have been employed as the second-line chemotherapy [9].

Molecular agents which target specific molecules or pathways are also available and used in the adjuvant setting [10]. A class of targeted agents used in ovarian cancer is PARP inhibitors, which are most effective for patients with germline or somatic mutations in *BRCA1/2* [11]. In both preclinical studies and translational studies of completed clinical trials, our group has recently reported that mutations in the tumor suppressor *TP53* predict the benefit of adding the antiangiogenic compound bevacizumab to chemotherapy upfront in advanced endometrial cancer [4,12]. However, for most patients, there are no genetic markers available to predict their response to commonly used agents [13]. Today, even as our knowledge of cancer genetics has increased dramatically, our ability to treat cancers based on the presence of genetic alterations is very limited. Particularly in gynecologic oncology, personalization of medical therapy is an unrealized goal [14].

Understanding patient and tumor genetic diversity and their influence on drug responses is an important step towards personalized medicine. In addition to research models to identify biomarkers of response to novel therapeutics or combinatorial regimens, we propose that patient-derived organoids (PDOs) hold great promise as valuable preclinical models which can provide insights into drug responses that are case-specific. Accumulating evidence indicates that PDOs can predict clinical outcomes in cancer patients [15,16,17]. Studies in several cancer types have established that PDOs recapitulate both the histologic and genomic features of the lesion from which they were derived [18,19,20]. Additionally, PDOs can grow with high efficiency in a short period of time which is much faster than generating a patient-derived xenograft (PDX model), enabling a priori prediction of responsiveness to chemotherapy, with the potential to substitute other regimens if primary resistance is demonstrated [21]. PDOs can also be tested for response to novel regimens including combinations of chemotherapy with targeted agents or multiple targeted agents which can be added to the patient’s initial round of therapy [22,23].

In this study, we describe progress on the generation of novel endometrial and ovarian cancer PDOs and drug testing. As an example of the power of PDOs to predict clinical outcomes, we present a case in which the PDO results reflected patient resistance to standard therapy, something we argue could be predicted upfront in the future using the PDO method for screening.

## 2. Materials and Methods

### 2.1. Clinical Features

All the studies were reviewed and approved by the University of Iowa’s IRB, protocol #201809807. The electronic medical record was reviewed to determine oncologic history, diagnosis, neoadjuvant and adjuvant treatment(s) and clinical responses. For patients who received platinum-based chemotherapy, platinum sensitivity was defined as progress-free survival of at least six months after the last platinum-containing therapy. Platinum resistance was defined as recurrence or persistence of disease within six months after the last platinum-containing treatment. Outcome data had not yet matured for some cases at the time of manuscript preparation; these cases are denoted “TBD” (to be determined). HER immunochemistry (IHC) was performed using clinical standards by the Central Pathology Laboratory at the University of Iowa, a CLIA-certified laboratory.

### 2.2. Generation of Patient-Derived Organoid Models Using Tumor and Normal Cells

Informed consent was obtained under the University of Iowa’s IRB protocol #201809807. Fresh tumor tissue was collected from each patient’s interval debulking surgery. Each patient’s ascites fluid, tumor and normal tissues were obtained, processed and cultured to create PDO cultures as previously described [20,23]; see additional details in the Supplementary Methods and Supplementary Figure S1. For H&E staining and IHC for HER2, organoids were exposed to 4% paraformaldehyde (PFA) for fixing cells and dissolving Matrigel. After pelleting by centrifugation, histologic gel was added to each sample. The samples were then transferred to a cassette, fixed in 70% ethanol overnight, sectioned and stained with either H&E or the HER2/neu antibody as for the biopsy and primary patient tumor specimens by the Central Pathology Laboratory at the University of Iowa.

### 2.3. Organoid Viability Assay

Viability of the tumor and normal organoids following drug treatment was performed as previously described [23]; see additional details in the Supplementary Methods. Briefly, organoids were collected with an organoid harvesting solution (Cultrex, R&D Systems, Minneapolis, MN, USA) and digested to single cells with TrypLE Express (Gibco, Waltham, MA, USA). Single cells were suspended in AdDE+++ medium with 10% Matrigel and seeded at a density of 10,000 cells/well in an ultra-low attachment 96-well U-bottom white plate. After 1–4 days, the organoids were exposed to carboplatin (1 µM), cisplatin (1 µM), paclitaxel (10 nM), bevacizumab (1 µM), gemcitabine (100 nM), topotecan (100 nM) or different combinations for 72 h at 37 °C. At the end of the incubation, an equal volume of the CellTiter-Glo 3D reagent (Promega, Madison, WI, USA) was added to each well and incubated for 25 min at room temperature. Luminescence, reflective of cell viability, was measured using a Gen5 Microplate Reader (BioTek, Winooski, VT, USA). All the tests were conducted in triplicate and the data were normalized to untreated controls (set at 100% viability).

### 2.4. Statistical Analysis

The data were analyzed using the GraphPad Prism software. Statistical significance of differences was determined using two-way ANOVA with the Greenhouse–Geisser correction and Tukey’s multiple comparison test, with individual variances computed for each comparison, or one-way ANOVA with Tukey’s multiple comparison test. All values are expressed as the mean  ±  standard deviation (SD) of at least three independent experiments unless otherwise indicated; * *p* < 0.05, ** *p* < 0.01, *** *p* < 0.001.

## 3. Results

### 3.1. Feasibility of Organoid Culture Creation from Freshly Resected Endometrial and Ovarian Cancer

At the time of each patient’s surgical debulking, ascites fluid, tumor and normal tissues were obtained, processed and cultured to create PDO cultures (see the Supplementary Methods and Supplementary Figure S1).

We successfully generated tumor organoid cultures from 43 of 52 tumor samples, an establishment success rate of 83% (Table S1). Successful cultures included 21 ovarian tumors, 22 endometrial tumors and nine normal organoids from normal uterine or fallopian tube samples. While the majority of models were generated using tumor tissues, seven ascites fluid samples were used to create PDOs; among those, six PDOs were generated. Successful growth of PDOs typically occurred within two–three days of surgical resection. In 12 cases with neoadjuvant therapy, six PDOs were generated, for the establishment success rate of 50%. In 40 non-neoadjuvant therapy cases, 37 tumor organoids were generated (success rate of 93%). We also observed that PDO generation using normal tissues was less successful than using tumor tissues, with the 33% establishment rate. The limited success rate may be due to the small size and the prevalence of stromal cells in the samples.

Next, drug screening was performed on 19 PDOs, a process that can be completed within 7–10 days from the time of surgery. Detailed information on the 19 models tested for drug sensitivity is summarized in Table 1.

### 3.2. Endometrial and Ovarian Cancer PDO Drug Response

Drug response assays were performed on 19 ovarian and endometrial cancer PDOs (Table 1) using therapeutic agents which are most commonly employed to treat patients with gynecologic cancer: carboplatin, paclitaxel, cisplatin, bevacizumab, gemcitabine and topotecan. PDO models that were created using ascites fluid samples are indicated with an * in Table 1.

**Figure 1:**
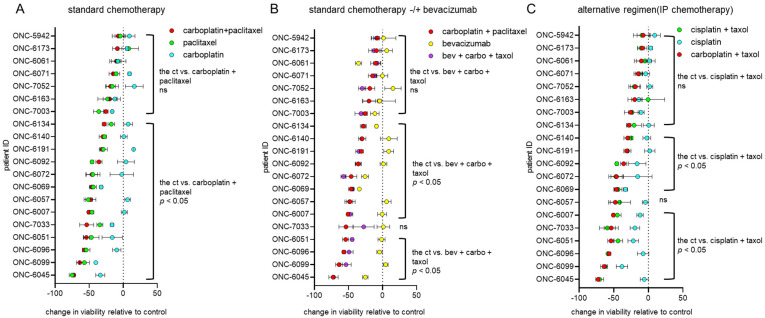
Therapeutic efficacy of the first-line therapies for endometrial and ovarian cancers using PDO models. Endometrial and ovarian cancer PDO response to (**A**) the standard chemotherapy, (**B**) the standard chemotherapy ± bevacizumab and (**C**) the most common alternative chemotherapy regimen. PDO models were treated with the indicated agents for 72 h, followed by assessment of cell viability. The data were calculated as the change in viability relative to the control which was set at 100% (i.e., no cell death). The samples are ordered based on increasing sensitivity to carboplatin + paclitaxel (red circles). Statistical significance was assessed by two-way ANOVA with the Greenhouse–Geisser correction and Tukey’s multiple comparison test. Significant differences vs. the control are annotated on each panel. All the statistical comparisons are provided in Supplementary Table S2, including specific *p*-values and comparisons of single agents vs. control or dual treatments.

Carboplatin, paclitaxel and their combination are the most frequently used in first-line chemotherapeutic regimens for gynecologic malignancies. PDO responses to these agents are shown in Figure 1A. As compared to the untreated controls, nine of the 19 PDOs exhibited a notable decrease in viability when treated with the combination of carboplatin and paclitaxel, with viability ranging from 46.1% to 72.7% after 72 h of treatment. In the combinatorial setting, the effects of the taxane, and not the platinum compound, appear to be the predominant driver of therapeutic effectiveness.

Bevacizumab is an antiangiogenic agent frequently used as an adjuvant therapy in gynecologic cancer [12,24,25,26]. Therefore, we also screened the PDOs for sensitivity to bevacizumab alone and in combination with the first-line standard chemotherapy. In general, bevacizumab as a single agent had only a modest impact on cell viability and little additional impact on cell killing when combined with chemotherapy (Figure 1B).

As an alternative to the first-line standard chemotherapy via the intravenous (IV) route, cisplatin combined with paclitaxel provides another option for patients to receive chemotherapy by the intraperitoneal (IP) route or via heated intraperitoneal chemotherapy [27,28]. In general, compared to the combination of carboplatin and paclitaxel, cisplatin combined with paclitaxel had similar impacts on cell viability (Figure 1C).

**Figure 2:**
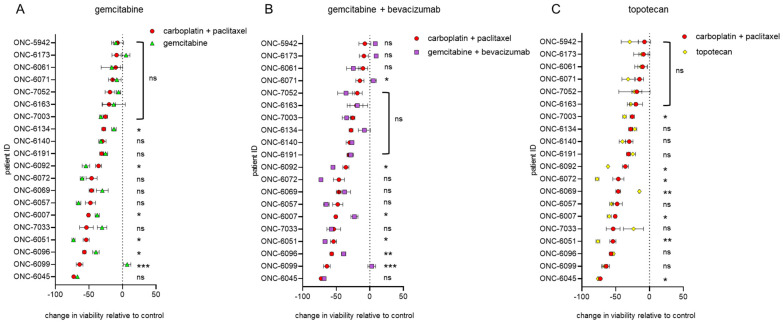
Therapeutic efficacy of common second-line therapies for endometrial and ovarian cancers using PDO models. Endometrial and ovarian cancer PDO response to second-line therapies: (**A**) gemcitabine, (**B**) gemcitabine + bevacizumab and (**C**) topotecan. PDO models were treated with the indicated agents for 72 h, followed by assessment of cell viability as in Figure 1. The data were calculated as the change in viability relative to the control, which was set at 100% (i.e., no cell death). Each panel includes response to the standard chemotherapy (carboplatin + paclitaxel, red circles) for comparison, and the samples are ordered based on increasing sensitivity to carboplatin + paclitaxel. Statistical significance was assessed by two-way ANOVA with the Greenhouse–Geisser correction and Tukey’s multiple comparison test. Significant differences vs. carboplatin + paclitaxel are annotated on each panel; ns: not significant; * *p* < 0.05; ** *p* < 0.01; *** *p* < 0.001. All the statistical comparisons are provided in Supplementary Table S2, including specific *p*-values and comparisons vs. the untreated control. Note that some cases have greater sensitivity to second-line treatments as compared to the standard chemotherapy, whereas others are more resistant. For example, ONC-6099 is relatively sensitive to chemotherapy but had no change in viability when exposed to gemcitabine (panel A).

Gemcitabine and topotecan are two commonly used second-line agents for cases of recurrent platinum-resistant gynecologic cancer [29,30,31,32,33]. Herein, we compared the effects of gemcitabine, topotecan and gemcitabine plus bevacizumab on cell viability. For some patients (ONC-6051, ONC-6072, ONC-6092), second-line therapies were more effective with respect to PDO cell killing than the first-line agents, but for most patients, the impact of those agents was not superior (Figure 2).

### 3.3. Proof-of-Concept: A PDO Model Reflects Treatment Response

#### 3.3.1. Patient Characteristics

**Figure 3:**
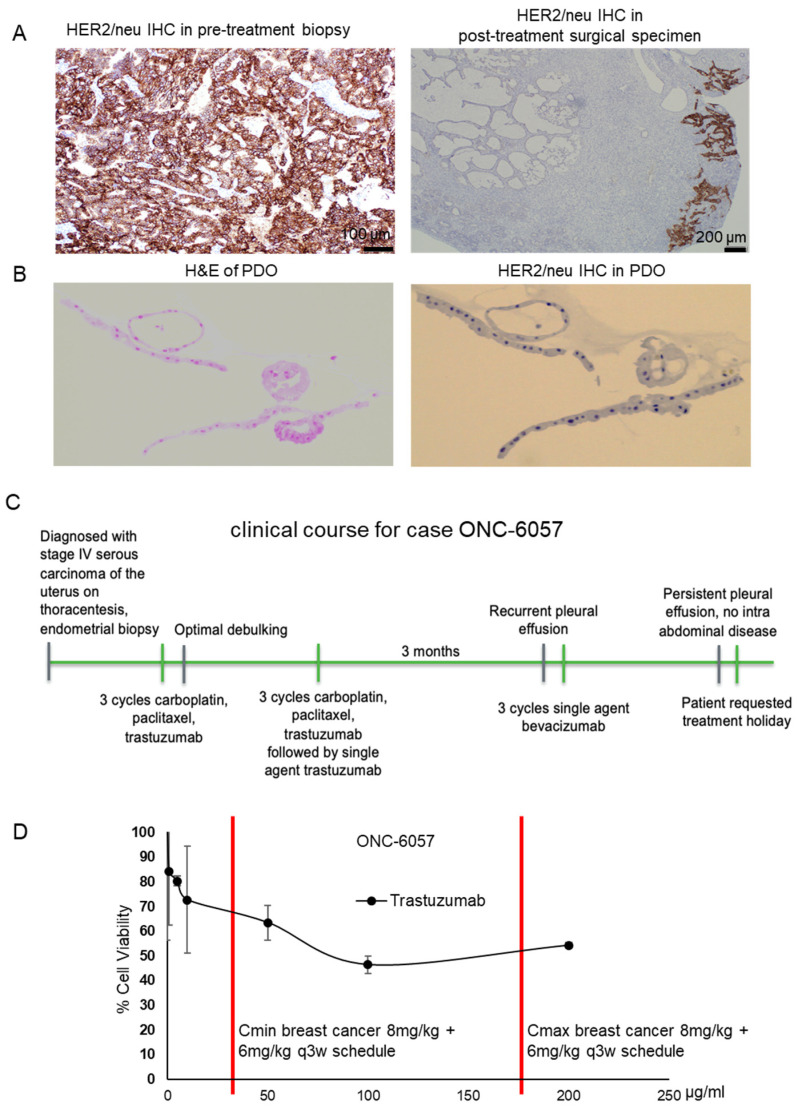
One advanced-stage high-grade serous endometrial carcinoma PDO model (ONC-6057) successfully predicted the patient’s clinical platinum and trastuzumab resistance. (**A**) HER2 IHC of the patient’s tumor obtained from the pretreatment biopsy and the posttreatment surgical specimen. Additional images are provided in Supplementary Figure S2. (**B**) H&E staining and HER2 IHC for the corresponding PDO model. Additional images are provided in Supplementary Figure S3. (**C**) Timeline of the patient’s clinical course. (**D**) PDO cells were treated with increasing concentrations of trastuzumab for 72 h, followed by assessment of cell viability. The data were calculated as the cell viability (%) relative to the untreated control which was set at 100%. The graph is annotated with the Cmin and Cmax values for trastuzumab used clinically in breast cancer. Note that an IC50 was not achieved with trastuzumab, even at concentrations greater than the Cmax.

Tissues were obtained from a 70-year-old woman who initially presented with shortness of breath, early satiety and postprandial right upper quadrant pain. Imaging was significant for a right pleural effusion, and the patient underwent a thoracentesis with temporary relief in symptoms. Cytology from her thoracentesis revealed PAX8-positive adenocarcinoma indicative of a gynecologic origin. Further imaging revealed multiple pulmonary emboli (PE), carcinomatosis, omental caking, ascites and a thickened endometrial stripe. Her history was negative for postmenopausal bleeding. An endometrial biopsy was obtained, which showed high-grade serous endometrial carcinoma, and HER2 immunostaining was positive (Figure 3A, pretreatment biopsy).

Given her new PE diagnosis and advanced-stage disease, the patient received three cycles of neoadjuvant carboplatin, paclitaxel and trastuzumab, a monoclonal antibody against HER2 (Figure 3B) [34]. Her CA125 decreased from 487 pre-treatment to 141 after the third cycle. Posttreatment imaging showed resolution of ascites, peritoneal carcinomatosis and omental cake. The patient subsequently underwent an exploratory laparotomy, total abdominal hysterectomy with bilateral salpingo-oophorectomy, infragastric and infracolic omentectomy, argon beam coagulation of tumor implants and optimal interval debulking. Final pathology from her debulking revealed her tumor to be of mixed serous and clear cell uterine histologies. Her postoperative course was uncomplicated, and she completed three more cycles of carboplatin, paclitaxel and trastuzumab with plans to continue maintenance trastuzumab until progression or toxicity. The patient received one cycle of maintenance trastuzumab, but before she was able to receive her second cycle (eighth overall), she experienced a rising CA125 and increasing shortness of breath. Imaging showed a recurrent pleural effusion, and a thoracentesis revealed recurrent adenocarcinoma.

Immunohistochemistry (IHC) performed on the cytology from this thoracentesis was estrogen receptor/progesterone receptor (ER/PR)-negative, suggesting that her pleural effusion was from the clear cell component of her cancer since the clear cell component of the primary tumor specimen was also negative for ER and PR. The patient then received three cycles of a single agent, bevacizumab, during which her CA125 continued to rise, and her dyspnea was stable. Repeat imaging showed no evidence of intraabdominal disease, but she had persistent pleural effusions. Due to poor tolerance of bevacizumab, the patient requested a chemotherapy holiday with as-needed thoracenteses.

#### 3.3.2. Trastuzumab Response in the Patient-Derived Tumor Organoid Model

This patient’s tumor was among the PDOs we created. We examined whether the tumor previously exposed to trastuzumab in the neoadjuvant setting before surgical resection would continue to demonstrate sensitivity to this agent or whether neoadjuvant treatment would select for resistant cell clones by the time surgery was performed. Thus, we assessed sensitivity of PDO cells to increasing concentrations of trastuzumab. Our results reflected potential resistance to trastuzumab based upon a lack of reaching an IC50 even at supraphysiologic concentrations (200 µg/mL) (Figure 3D). The lack of response to trastuzumab was also predicted by the lack of HER2 immunostaining in the postsurgical specimen and the organoid sample (Figure 3A,B). Pathological analysis of the postsurgical specimen indicated some HER2 immunoreactivity in the cystically dilated glandular areas (Figure 3A, right margin of the posttreatment surgical specimen), whereas the stromal region and clear cell component (left side of the posttreatment surgical specimen) were devoid of HER2.

**Figure 4:**
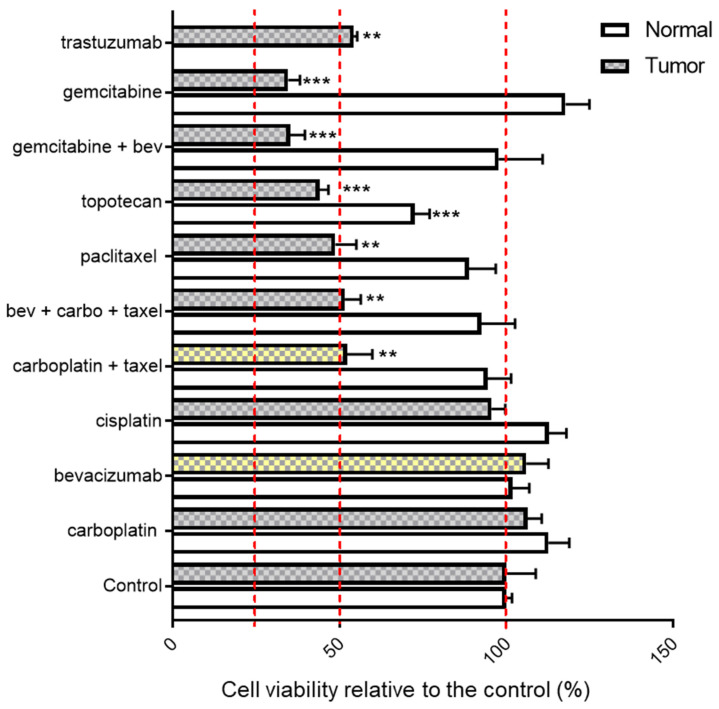
Varying drug sensitivity to the standard and alternative therapeutic options for the ONC-6057 PDO. Waterfall plot of the patient’s (ONC-6057) tumor organoid drug sensitivity with the paired normal uterine drug sensitivity. The cytotoxic agents in the chemotherapy regimen (carboplatin + paclitaxel and bevacizumab) the patient received after resistance to trastuzumab was observed are highlighted in yellow. The change in viability was calculated relative to the control which was set at 100% (i.e., no cell death). Statistical significance was assessed by ordinary one-way ANOVA with Tukey’s multiple comparison test; ** *p* < 0.01, *** *p* < 0.001 vs. the paired control (normal or tumor specimen).

To further assess which agents might be therapeutically effective, the PDO was screened for sensitivity to alternative agents (Figure 4). The patient’s PDO model showed a clear lack of response to platinum compounds with no change in cell viability after treatment with carboplatin or cisplatin as compared to the untreated control. Furthermore, the PDO was not sensitive to bevacizumab (105.9% viability vs. the control), the agent the patient received after failing trastuzumab. Organoid cells were more susceptible to paclitaxel, with 48.7% cell viability after exposure to this single agent. The patient’s PDO was also more sensitive to gemcitabine (65% decrease in viability) and topotecan (56% decrease in viability) as compared to the chemotherapy regimen previously received, carboplatin + paclitaxel.

We also assessed drug sensitivity using a subset of agents in normal tissue organoids from the same patient (Figure 4). Gemcitabine, topotecan and paclitaxel, which induced more than 50% cell death in tumor organoids, all resulted in minimal cell killing of organoids derived from non-malignant tissues.

## 4. Discussion

We intuitively know that each patient’s tumor is different, with potentially unique genetic and phenotypic characteristics. It is becoming increasingly clear that the conventional way to treat patients using a limited panel of first-line agents may miss important opportunities to optimize therapy. In contrast, personalized medicine strives to match the unique patient and tumor characteristics with innovative treatment regimens tailored to each case; however, progress in implementing precision therapies has been hampered by the lack of sufficient biomarkers that predict response to each therapeutic agent. One way to improve outcomes without the long and laborious process of biomarker identification and validation is to utilize tumor cells themselves as a patient avatar.

PDO culture technology is a novel and powerful tool for generating and maintaining three-dimensional patient tumor and normal tissues in vitro [35]. Since the time from surgical resection to drug testing results takes 7–10 days, use of organoids from primary patient tissues enables the testing of a range of compounds for efficacy even before clinical treatment decisions must be made. In addition, the establishment of organoids from non-malignant tissues from the same patient provides an opportunity to test potential drug toxicities. The methods reported in this study as well as the results reported by others [36] strongly support the concept that establishment of PDOs from patients under treatment for gynecologic cancers is feasible and potentially useful to understand the impact of therapeutic agents on cell viability in a time frame that is congruent with making actionable treatment decisions [37,38]. We propose that testing PDOs for therapeutic response is a methodology worthy of expansion as a means to achieve precision medicine in clinical practice.

Overall, our success rate in generating PDOs was quite high. Among the nine cases which failed to culture, two thirds were from the patients who received neoadjuvant chemotherapy. Neoadjuvant therapy has been shown to negatively affect the success rate of PDO generation [39]. We speculate the reason why tumor tissues from the patients who received neoadjuvant chemotherapy failed to generate organoids is because the tumors may already be undergoing chemotherapy-induced cell death. Accordingly, after we collected the tumor and isolated cells from the tumor, cell viability was too low for organoid culture. In addition, for the vast majority of tissues that were not successfully cultured, we observed that the time from surgical removal to culture was greater than 45 min, though some PDO models were successfully cultured after a delay of several hours. Nonetheless, mitigating the delay from surgical excision to tissue processing will undoubtedly improve the success rate of PDO creation. Other variables include the sample size and the percentage of viable cells within the specimen.

To illustrate how PDO data could be used in the future to predict clinical outcomes, tumor and normal PDOs were established from a patient with advanced high-grade serous endometrial carcinoma. Drug sensitivity testing reflected resistance to all agents given in the neoadjuvant setting. This raises the possibility of tumor evolution after initial exposure to drugs and brings into question the continued use of agents used in the neoadjuvant setting for ongoing adjuvant treatment, i.e., after surgical removal of the tumor. We propose that drug sensitivity testing of PDOs may provide more insight into which agents are most effective at later timepoints. This is based on our proof-of-concept data that the PDO model successfully predicted the patient’s clinical platinum and trastuzumab resistance despite her initial response to neoadjuvant chemotherapy.

Although PDOs have proved useful for many applications in basic research and contributed to biomedical advances [40], the road to real-life applications is long, with many technologies to be explored in this area. PDOs have not yet been widely used for translational studies yet due to some limitations [41]. For example, compared with cancer cell lines, organoid culture consumes more time and resources [40]. For most models we created, the amount of tumor tissue we received from the operating room was quite small, and we prioritized drug screening experiments. This precluded extensive molecular characterization for each model, such as comparison of biomarkers between the primary tumor and the PDO model. Given the potential for evolution with prolonged culture, we performed the bulk of experiments on passage 1–2 samples rather than expanding them to get enough cells for additional analyses. While we stored some organoids in liquid nitrogen, we found that the growth rate is significantly slower after cryopreservation. Another limitation is PDO culture may not reflect the tumor environment due to lack of stroma, blood vessels and immune cells [42]. Incomplete representation of intratumoral heterogeneity is another limitation for PDO culture. Many studies have proved that PDOs can retain the heterogeneous genetic composition of the primary tumor [43,44]. However, each PDO is derived from a small part of the tumor and may not reflect different cell populations of the primary tumor. For example, Roerink et al. have shown the extensive genetic diversification in organoids derived from different regions of the same tumor using comprehensive genetic analysis [45]. Despite these limitations, organoids have emerged as a physiologically relevant ex vivo model to study cancer. In addition, there are several co-clinical trials ongoing in different cancer types that compare drug responses in PDOs to corresponding patient outcomes (e.g., NCT03979170, NCT04859166, NCT04555473).

In conclusion, PDOs provide a valuable preclinical model system to support new opportunities for personalized medicine. Our results demonstrate, first, that PDOs can be established in a high percentage of cases; second, that drug testing on PDOs can be performed in a timely manner and that PDOs have the potential to identify agents with the greatest efficacy in each individual case. This is an important first step in establishing a feasible functional treatment response platform to help more patients with gynecologic cancer receive precision therapeutic care.

## Figures and Tables

**Table 1:** Patient and tumor characteristics corresponding to the 19 PDOs used for drug screening studies. IV: intravenous; IP: intraperitoneal; NED: no evidence of disease; N/A: not available; TBD: to be determined; * indicates the PDOs that were generated using the ascites fluid samples.

Patient ID		Cancer Type	Stage	Neoadjuvant Treatment	Adjuvant Treatment	Platinum-Sensitive?	Disease Status
Ovarian Cancer	ONC-5942	High-grade serous	IVB	Six cycles of carboplatin and paclitaxel followed by two cycles of carboplatin	Three cycles of doxorubicin and bevacizumab followed by bevacizumab	No	NED
	ONC-6007 *	High-grade serous	IVB	No	Six cycles of IV/IP cisplatin and paclitaxel	Yes	Alive with disease
	ONC-6045 *	High-grade serous	IIIB	No	One cycle of a single agent, carboplatin, followed by six cycles of carboplatin and paclitaxel	Yes	NED
	ONC-6069	High-grade serous	Recurrent	Six cycles of carboplatin and paclitaxel followed by olaparib	Olaparib followed by gemcitabine	No	Dead of disease
	ONC-6072	High-grade serous	IC	No	None	N/A	Lost to follow-up
	ONC-6134	High-grade serous	IIIA	No	Six cycles of carboplatin and paclitaxel	Yes	NED
	ONC-6163	High-grade serous	Recurrent	No	Six cycles of carboplatin and doxorubicin followed by olaparib	TBD	NED
	ONC-7052	High-grade serous	IIIC	No	Six cycles of IV/IP cisplatin and paclitaxel	TBD	NED
	ONC-6061	Low-grade serous	IIIC	No	Two cycles of carboplatin and paclitaxel followed by seven cycles of a single agent, carboplatin, followed by letrozole	No	Alive with disease
	ONC-7063	Clear cell	IC1	No	Three cycles of carboplatin and paclitaxel	TBD	NED
	ONC-6092	Clear cell	IC	No	Three cycles of carboplatin and paclitaxel	Yes	NED
Endometrial Cancer	ONC-6096	Endometrioid, grade 1	IA	No	Observation	N/A	NED
	ONC-6191	Endometrioid, grade 1	IB	No	N/A	N/A	NED
	ONC-6051	Endometrioid, grade 2	IA	No	Observation	N/A	NED
	ONC-6173	Endometrioid, grade 2	IA	No	Observation	N/A	NED
	ONC-6071	Endometrioid, grade 3	IA	No	Observation	N/A	NED
	ONC-6057	Serous	IVB	Three cycles of carboplatin, paclitaxel and trastuzumab	Three cycles of carboplatin, paclitaxel and trastuzumab followed by a single agent, bevacizumab	No	Alive with disease
	ONC-6099	Serous	IVB	Six cycles of carboplatin and paclitaxel	Three cycles of paclitaxel and bevacizumab	No	Dead of disease
	ONC-7003	Mixed serous/endometrioid	IA	No	Patient declined adjuvant therapy	N/A	NED

*: PDO models that were created using ascites fluid samples.

## Data Availability

All the data generated or analyzed in this study are provided in either the main manuscript or as Supplementary data.

## Supplementary Materials

The following are available online at https://www.mdpi.com/article/10.3390/cancers13122901/s1, Supplementary Methods: Protocol for Organoid Generation, Figure S1: Schematic presentation of procedures for the organoid culture of endometrial and ovarian cancer cells derived from patient tumors, Figure S2: Pretreatment biopsy specimen for ONC-6057, Figure S3: Additional H&E and HER2 IHC images of the ONC-6057 PDOs, Table S1: Characteristics of all the specimens attempted to be cultured as PDO models, including the paired normal tissue specimens, Table S2: Statistical analyses for drug screening in Figure 1 and Figure 2.
