# Supplementary material for: Successful Patient-Derived Organoid Culture of Gynecologic Cancers for Disease Modeling and Drug Sensitivity Testing
Source: Cancers (Basel). 2021 Jun 10;13(12):2901. doi: 10.3390/cancers13122901 (PMC8229222; doi:10.3390/cancers13122901)
Supplement: Supplementary file 1 [file cancers-13-02901-s001.zip › Supplemental Figures.pdf]

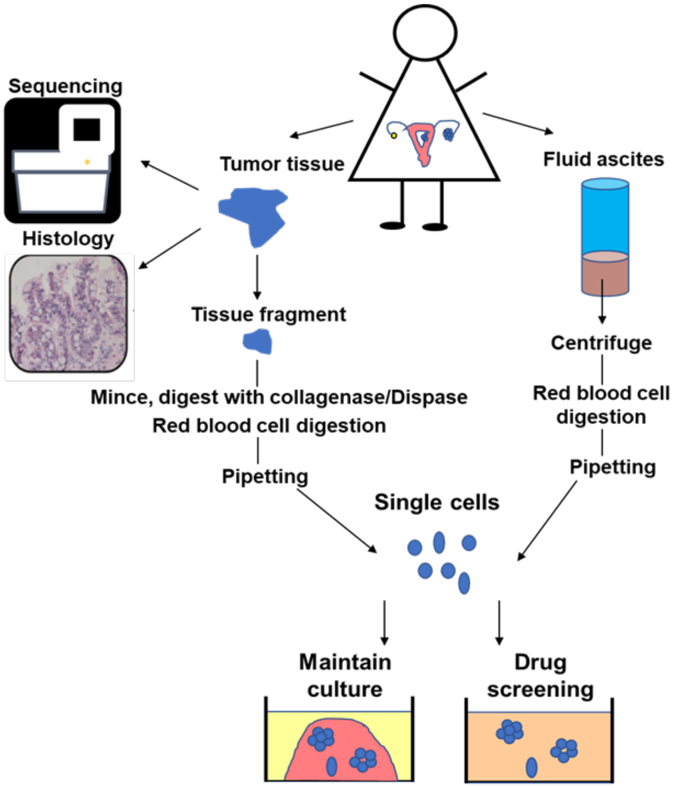

Supplemental Figure S1. Schematic presentation of procedures for the organoid culture of endometrial and ovarian cancer cells derived from patient tumors.

Pre-treatment biopsy specimen

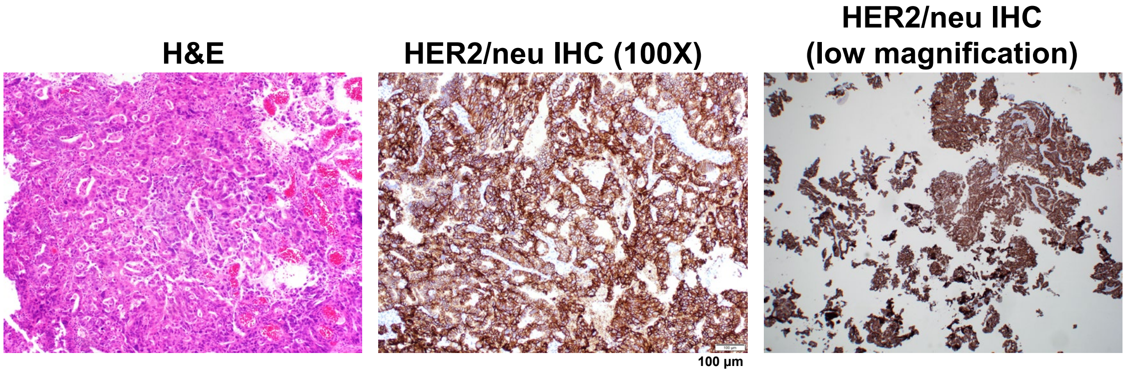

Supplemental Figure S2: Pretreatment biopsy specimen for ONC-6057.

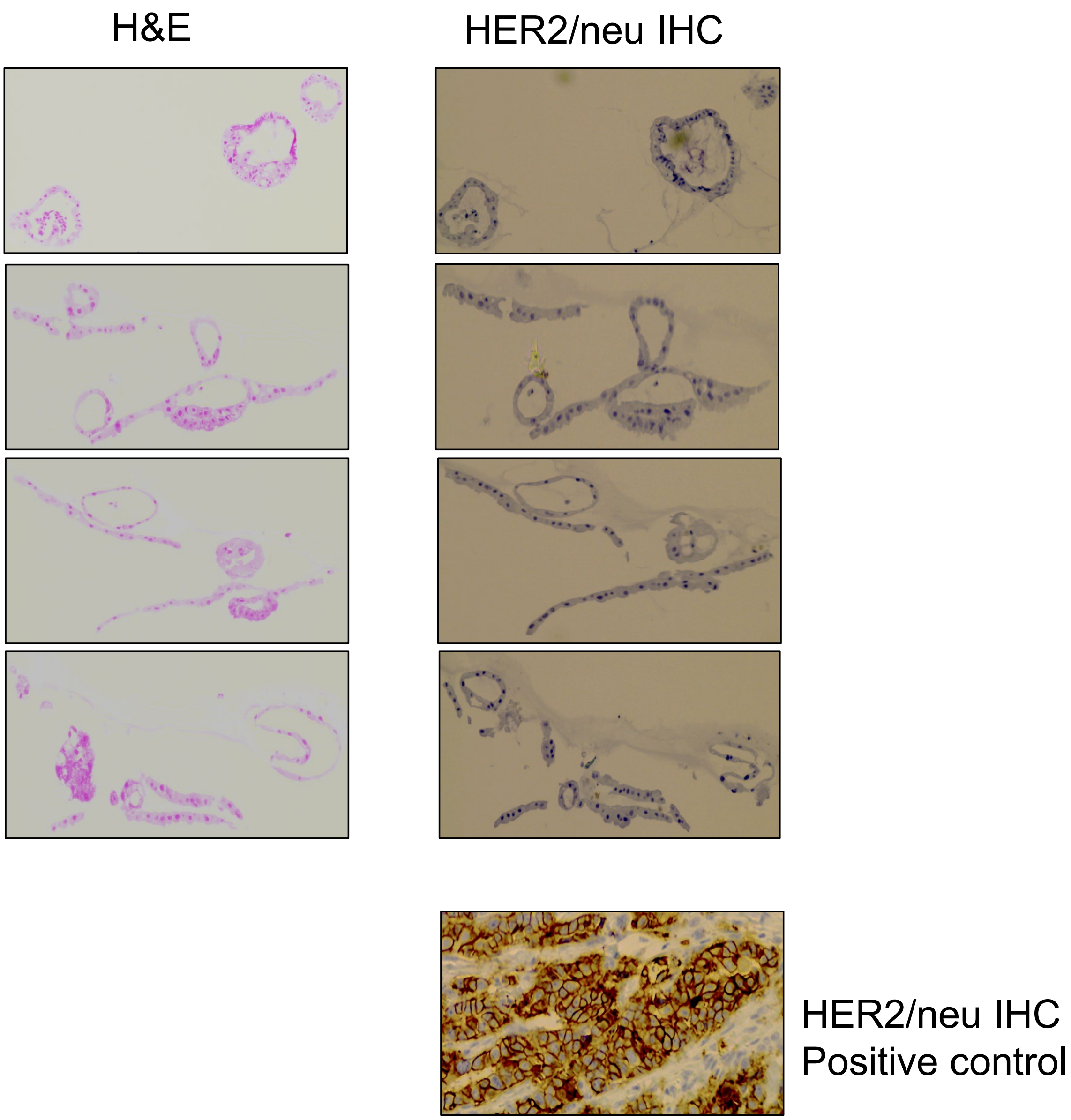

Supplemental Figure S3: Additional H&E and HER2 IHC images of ONC-6057 PDOs. Also included is a positive control for HER2/neu IHC, which contains robust membrane staining of HER2.
