# Supplementary material for: Successful Patient-Derived Organoid Culture of Gynecologic Cancers for Disease Modeling and Drug Sensitivity Testing
Source: Cancers (Basel). 2021 Jun 10;13(12):2901. doi: 10.3390/cancers13122901 (PMC8229222; doi:10.3390/cancers13122901)
Supplement: Supplementary file 1 [file cancers-13-02901-s001.zip › Supplemental Methods.pdf]

cancers MDPI

Supplemental Methods

Successful Patient Derived Organoid Culture of Gynecologic Cancers for Disease Modeling and Drug Sensitivity Testing

Jianling Bi, Andreea M. Newtson, Yuping Zhang, Eric J. Devor, Megan I. Samuelson, Kristina W. Thiel and Kimberly K. Leslie

Protocol for Organoid Generation

1. Materials and Preparation:

1) Digest buffer: 2U/ml Dispase Ⅱ,1mg/ml collagenase P, 5μM Y27632 and 10μg/ml Dnase I in advanced DMEM-F12 media (prepared fresh every experiment)
2) AdDF+++ media: Advanced DMEM-F12 with 1X Glutamax,10 mM HEPES and Pen strep
3) Red blood cell lysis buffer (Roche)
4) Culture media: AdDF+++ media supplemented with 2% Primocin (InvivoGen, San Diego, CA), 1:50 B27 supplement (Life Technologies, Paisley, UK), 1.25mM N-acetyl-l-cysteine (Sigma-Aldrich, St. Louis, MO, USA), 250 ng/ml R-spondin1 (R&D Systems, Minneapolis, MN), 100 ng/ml Noggin (R&D Systems), 5 mM nicotinamide (Sigma), 50 ng/ml recombinant human EGF (R&D Systems), 100 ng/ml recombi-nant human FGF10 (R&D Systems), 10μM forskolin (R&D Systems), 5μM A8301 (Tocris Bioscience, Avonmouth, Bristol, UK), 500 ng/ml hydrocortisone (Sigma-Aldrich), 37.5 ng/ml Heregulinβ-1 (R&D Systems),100nM β-Estradiol (Sigma-Aldrich) and 10μM Y27632 (Tocris BioscienceK)
5) Pre-warm the 24- and 96-well U-bottom white plates in the incubator.
6) Primary human gynecologic tissue samples from surgical staff are obtained in a sterile vessel (e.g., 50-ml conical tube) containing advanced DMEM-F12 media. Ascites fluid is collected in a sterile container.

2. Procedure:

2.1. Processing of tumor and normal tissue

1) Using sterile forceps place the ovarian and endometrial tumor and normal tissue in a 100mm plate with AdDF+++ media and wash tissue with cold PBS to remove blood and fat.
2) Cut the tissue into 2-3 mm pieces.
3) Put the small pieces of tissue in a 50 ml conical tube with digest buffer, swirl tubes every 10 minutes or place in a gently rotating incubator for a total incubation time of 0.5h-1h at 37°C.
4) When the tissue becomes visibly smaller, use a 1ml pipet tip and pipet up and down several times to break up tissue, followed by a 10ml syringe with 23 g needle.

5) Filter the mixture through a 40 μm cell strainer and centrifuge at 500 x *g* for 10 min.
6) If samples have blood cells, incubate with blood cell digest buffer for 5 min at room temperature, followed by centrifugation at 500 x *g* for 5 min. Wash the cell pellet with AdDF+++ media and centrifuge at 500 x *g* for 5 min to collect the cells.
7) Resuspend the cell pellet with culture media and calculate the cell number. The number of cells to be plated can be determined as follows:

A) Culture of organoids in 24 well plate: To plate a 24-well plate, the desired number of cells is $1x10^5$ cells per well in 50 μl Matrigel (Corning) per well. Resuspend cells with cool Matrigel and seed into the middle of the well by gently pipetting 50 μl into the center of each well. Incubate plates for 15 min in 37°C, then add 500 μl culture media per well on top of the Matrigel. Organoids are maintained in humidified incubators at 37°C with 5% CO_2.
B) Drug screening: Drug screening is performed in 96-well U-bottom white plates (Corning), with $1x10^4$ cells per well. Resuspend cells in Matrigel+culture media to achieve a final concentration of Matrigel of 10% and 50 μl total volume per well. Always set up a "parallel" 24 well plate to visually confirm by microscopy that organoids have formed prior to drug screening (1-4 days). For treatments, an additional 50 μl of culture media containing the experimental agents at a 2X concentration are added to each well (the total volume = 100 μl). Cell viability is assessed using Cell Titer Glow 3D Reagent (Promega).
For small tissue samples in which there are not enough cells to seed a 24-well plate and a 96-well plate for drug screening, cells can be expanded in a 24 well plate and passaged to more plates after organoid formation to reach a higher density. To transfer expanded cells to 96-well plates, remove culture media and wash organoids with PBS. To break down the Matrigel, incubate with organoid harvesting solution (Cultrex) per the manufacturer's instructions; best results are obtained with constant gentle agitation of the plates. Transfer the contents of each well to a 15-ml conical tube (pool all wells). To digest organoids to single cells, incubate with 3ml TrypLE Express (Gibco, USA) supplemented with 10 μg/ml DNAse I and 10.5μM Rho Kinase inhibitor (Y-27632) at 37°C for 10-15 min in a rotating incubator. Break up the organoids to single cells with 100 μl pipet tip and pipet up and down. Single cells can be suspended in culture media with 10% Matrigel and as above.

2.2. Processing of ascites fluid

1) Transfer 50–100 ml of ascites fluid from the sterile container to 50 ml conical tubes.
2) Centrifuge at 500 x *g* for 10min at 4 °C, remove the supernatant, add 2 ml blood cell lysis buffer for 5 min, centrifuge at 500 x *g* for 5min at 4 °C, then wash with 1x cold PBS, centrifuge again at 500g for 5 min at 4 °C, remove the supernatant and wash with AdDF+++ media.
3) Carefully discard supernatant with a 5 ml pipet.
4) Resuspend cells in culture media as in Step 2.1.7, then following the protocol in 2.1 for plating cells.
