# Supplementary material for: Successful Patient-Derived Organoid Culture of Gynecologic Cancers for Disease Modeling and Drug Sensitivity Testing
Source: Cancers (Basel). 2021 Jun 10;13(12):2901. doi: 10.3390/cancers13122901 (PMC8229222; doi:10.3390/cancers13122901)
Supplement: Supplementary file 1 [file cancers-13-02901-s001.zip › Supplemental Table S1.pdf]

Table S1. Characteristics for all specimens attempted to be cultured as PDO models, including paired normal tissue specimens. * indicates PDOs that were generated using ascites fluid samples.

	Patient ID	Cancer Type	Neoadjuvant Treatment	Stage	Successful Culture of Tumor PDO	Normal Tissue Type	Successful Culture of Normal PDO
Ovarian	ONC-5942	High-grade serous	Y	IVB	Y	-	
	ONC-6007	High-grade serous	N	IVB	Y*	-	
	ONC-6045	High-grade serous	N	IIIB	Y*	-	
	ONC-6069	High-grade serous	Y	Recurrent	Y	Ovary	N
	ONC-6072	High-grade serous	N	IC	Y	Ovary	N
	ONC-6070	High-grade serous	Y	IVB	N	Ovary	N
	ONC-6088	High-grade serous	N	IIA	Y	-	
	ONC-6134	High-grade serous	N	IIIA	Y	-	
	ONC-6163	High-grade serous	N	Recurrent	Y	Ovary	N
	ONC-6562	High-grade serous	N	IVA	Y	Ovary	Y
	ONC-6663	High-grade serous	N	IIIC	Y	-	
	ONC-6683	High-grade serous	Y	IVB	Y	Fallopian tube	Y
	ONC-6693	High-grade serous	Y	IIIC	N	Fallopian tube	N
	ONC-6695	High-grade serous	N	IC1	Y	Fallopian tube	Y
	ONC-7033	High-grade serous	N	IVA	Y*	-	
	ONC-7052	High-grade serous	N	IIIC	Y*	-	
	ONC-7059	High-grade serous	Y	At least IIB	N	-	
	ONC-7077	High-grade serous	Y	IIIC	N	Ovary	N
	ONC-7253	High grade serous	N	IIIC	Y*	-	
	ONC-7183	Low-grade serous	Y	IIIC LGS	N	-	
	ONC-6497	Low-grade serous	Y	IV	Y	-	
	ONC-7017	Low-grade serous	Y	IVB	N	-	
	ONC-6061	Low-grade serous	N	IIIC	Y	Fallopian tube	N
	ONC-7063	Clear cell	N	IC1	Y	-	
	ONC-5945	Clear cell	N	IC	Y	-	
	ONC-6092	Clear cell	N	IC	Y	-	

	ONC-0000	Carcinosarcoma	N	IVA	Y	-	
	ONC-7234	Malignant brenner tumor	N	IIB	N*	-	
**Uterine**	ONC-5768	Endometrioid, Grade 1	N	IIIC2	Y*	-	
	ONC-6096	Endometrioid, Grade 1	N	IA	Y	Epithelial endometrium	N
	ONC-6191	Endometrioid, Grade 1	N	IB	Y	Epithelial endometrium	N
	ONC-6692	Endometrioid, Grade 1	N	IA	Y	Epithelial endometrium	N
	ONC-6790	Endometrioid, Grade 1	N	IA	Y	Epithelial endometrium	N
	ONC-6818	Endometrioid, Grade 1	N	IA	Y	Epithelial endometrium	Y
	ONC-6844	Endometrioid, Grade 1	N	IA	Y	Epithelial endometrium	Y
	ONC-6885	Endometrioid, Grade 1	N	IA	Y	-	
	ONC-6932	Endometrioid, Grade 1	N	IA	N	Epithelial endometrium	N
	ONC-7076	Endometrioid, Grade 1	N	IA	Y	Epithelial endometrium	N
	ONC-7026	Endometrioid, Grade 2	N	IA	Y	-	
	ONC-6051	Endometrioid, Grade 2	N	IA	Y	-	
	ONC-6173	Endometrioid, Grade 2	N	IA	Y	Epithelial endometrium	N
	ONC-6498	Endometrioid, Grade 2	N	IA	Y	-	
	ONC-6071	Endometrioid, Grade 3	N	IA	Y	Epithelial endometrium	N
	ONC-6925	Endometrioid, Grade 3	N	IA	Y	Epithelial endometrium	N
	ONC-6223	High Grade	N	IIIC2	N	-	
	ONC-6057	Serous	Y	IVB	Y	Epithelial endometrium	Y
	ONC-6099	Serous	Y	IVB	Y	-	
	ONC-6720	Serous	N	IIIC1	Y	Epithelial endometrium	Y
	ONC-6373	Serous	N	IIIA	Y	Epithelial endometrium	N
	ONC-6560	Clear cell	N	IA	Y	Epithelial endometrium	Y
	ONC-6742	Mixed serous and endometrioid	N	IA	Y	Epithelial endometrium	N
	ONC-7003	Mixed serous and endometrioid	N	IA	Y	Epithelial endometrium	Y
